# Research on the Influence of Education of Farmers’ Cooperatives on the Adoption of Green Prevention and Control Technologies by Members: Evidence from Rural China

**DOI:** 10.3390/ijerph19106255

**Published:** 2022-05-20

**Authors:** Lei Luo, Dakuan Qiao, Ruixin Zhang, Chenhao Luo, Xinhong Fu, Yuying Liu

**Affiliations:** 1 School of Management, Sichuan Agricultural University, Chengdu 611130, China; luolei@stu.sicau.edu.cn (L.L.); 2020309003@stu.sicau.edu.cn (D.Q.); zhangrx@stu.sicau.edu.cn (R.Z.); 2 School of Accounting, Southwestern University of Finance and Economics, Chengdu 611130, China; jasonluo8878@foxmail.com; 3 Sichuan Rural Development Research Center, Chengdu 611130, China

**Keywords:** education of farmers’ cooperatives, cognition of green prevention and control technologies, adoption of green prevention and control technology, intermediary effect

## Abstract

The study explores the impact of education of farmers’ cooperatives on members’ green production behavior. The Probit, Oprobit model and the mediation effect model are used to analyze the influence mechanism of the cooperative’s education on the members’ adoption of four types of green prevention and control technologies and the overall adoption rate, and the instrumental variable method is used for endogeneity treatment and robustness test. The results show that: (1) The education of cooperatives have a significant positive impact on the members’ physical pest control technology, biological pesticide application technology, water and fertilizer integration technology, scientific pesticides reduction technology, and the overall adoption rate plays a critical role. As a result, there is a certain degree of heterogeneity in different intergenerational member groups. (2) The education of cooperatives can significantly enhance members’ cognition of green prevention and control. (3) Through on-the-spot demonstration and general meetings of the members to carry out education, members are more likely to adopt green prevention and control technologies. These findings shed light on the mechanisms by which cooperative’s education affect the green production behavior of cooperative members and provide important policy implications for green agricultural development.

## 1. Introduction

China is implementing the strategy of rural revitalization, and the development of green agriculture is an important part of it. However, the average usage of chemical pesticides per unit area in China is 2.5 times that of developed countries [1]. Excessive use of chemical pesticides is one of the main culprits that aggravate China’s agricultural non-point source pollution and lead to the degradation of agricultural ecosystems [2]. At the China Rural Work Conference 2020, President Xi Jinping stressed the need to “promote the prevention and control of agricultural non-point source pollution with the spirit of nailing the nails”. In 2021, China formulated the National Agricultural Green Development Plan during the 14th Five-Year Plan, which clearly stated that by 2025, the agricultural ecosystem will be significantly improved, the supply of green products will be significantly increased, and the capacity of emission reduction and carbon sequestration will be significantly enhanced. In order to achieve the goal of green agricultural development, China is committed to promoting the Chinese practice of integrated pest management (IPM)-Green prevention and control technologies (GCT) [3]. It is a complex technology set, mainly including four categories: physical and chemical induction and control technology, biological control technology, ecological regulation technology, and scientific pesticides use technology, combined with the “Technical Regulations for Green and Efficient Production of Late-Mature Citrus”. At present, the application level of GCT in China is not high, which is one of the “bottleneck problems” restricting the sustainable development of China’s agriculture [4]. The GCT coverage of major crops in China reached 27.2%, but there is still a long way to go before the goal of covering more than 50% of major crops in 2022 [5].

There are abundant research on farmers’ GCT adoption in academic circles. It is generally believed that GCT adoption is related to farmers’ age [6], education level [7], geographical environment [8], farm size [9], income [10], labor force [11], etc. Moreover, GCT adoption is also related to external factors such as government regulation [12], technology integration and application [13], technical training [14], market supervision, and docking [15]. However, agricultural non-point source pollution is hidden, scattered, and difficult to find [16]. On the premise of not changing the individual’s own awareness and concept of green prevention and control, the effect of external factors constraining farmers to adopt green technologies is often unsatisfactory [17]. It is worth noting that education can strengthen farmers’ subjective awareness of ecological environment protection [18]. Education, as the benchmark orientation for the adoption decision of agricultural green prevention and control technology [19], can make farmers to improve the cognition of agricultural green control, green control advantages and disadvantages of traditional control [20], so that farmers can establish the concept of green environmental protection at the subjective level and effectively promote the adoption of GCT [21]. At the same time, Elahi et al. found that the education of green technology led by the government costs a lot of money and was ineffective, and it was difficult for the government to form a scale effect. Therefore, agricultural technology extension institutions such as farmers’ cooperatives should be encouraged to carry out education and training to promote the adoption of environmentally friendly technology by farmers [22]. Since education are inseparable from the trust between organizations and individuals [23], mutual understanding between publicists and farmers [24], consideration of farmers’ individual needs [25], and the convenience of information acquisition [26], farmers cooperatives, as spontaneous farmers’ associations deeply embedded in the rural social network [27], meet the educational needs of the farmers mentioned above. The International Cooperative Alliance (ICA) established the Manchester Principles in 1995: cooperatives must develop the cause of “education, training and information”, therefore, farmers’ cooperatives have an important function that cannot be ignored [28]. Farmers’ cooperatives can take advantage of geography and organization to carry out in-depth information and training on green prevention and control in rural society, promote the general members to understand GCT, and transform the GCT adoption process from “I want to use it” to “I want to know how to use it”, so as to improve the subjective initiative of adopting GCT. Some studies have shown that the embeddedness of cooperatives can promote members’ willingness to use green production technologies [29], making it easier for them to adopt GCT [30]. However, there is no empirical analysis on whether the farmers’ cooperatives have trained staff to promote the adoption of GCT, and its influence mechanism has not been verified.

Previous studies have found that individuals’ cognition of GCT is a key factor affecting their adoption of production technologies [31], which means that with a deeper understanding of GCT, individuals may increase the possibility of adopting GCT [32]. Due to the wide scope of cognition, the division of value cognition in academic circles has not been unified, but the most influential one is that Stern et al. proposed that the generation of pro-environmental behavior stems from three basic cognitive orientations: self-interest care, ecological care, and altruistic care [33]. Based on the cognitive orientation of Stem and the characteristics of green prevention and control technology, the cognition of members in this paper is divided into three aspects: economic cognition (self-interested concern), environmental cognition (ecological concern), and social cognition (altruistic concern). The education of cooperatives can effectively enhance members’ cognition and understanding of the economic, environmental, and social benefits brought by green prevention and control technology [34]. When individual members form differentiated cognition levels, the equilibrium point of resource allocation that maximizes their benefits may change, leading to differences in behavioral intentions, which in turn determine their adoption decisions [35], and members’ cognition may play an intermediary role in this process.

The main contributions of this paper are as follows. First, different from existing research that only focuses on government education, this paper empirically tests whether the cooperatives have sufficient support to increase adoption of GCT by members, and solves the endogeneity problem through instrumental variables. Second, based on the existing research, this paper analyzes the influence mechanism of cooperatives’ education on the GCT adoption of members, and explains the mediating role of members’ cognition in it. Therefore, this study provides a new perspective on solving the difficulties of expanding the use of GCT that results from citrus farmers.

## 2. Theoretical Analysis

Any adoption decision is based on information acquisition [36]. Persuasion theory believes that by disseminating a certain aspect of information, individuals will deepen their understanding of this aspect of knowledge [37], and when they have enough of this information, they will often persuade themselves to actively participate, and then affect the individual’s related willingness and decision-making. Training farmers is the main way to disseminate GCT operational knowledge, which can promote people to master the GCT operating points skillfully, and then generate the willingness to adopt technology [38]. Cooperatives have the natural advantage of playing a training function in rural society, and are an important organizational carrier for information transmission [27]. In practice, cooperatives can effectively carry out targeted and can provide targeted technical information for farmers to implement newer green prevention technology [39], and reduce the marginal cost of technology adoption [40]. At the same time, cooperatives can use institutional advantages such as member meetings to guide members to communicate experiences in GCT, and form a stable rural eco-environmental social circle. By systematic sharing of information between members, they can become familiar with the structure and process of GCT, thereby promoting the adoption of GCT by members [28]. Theoretically, the cooperatives can provide a more efficient way of transmitting information which can make members more willing to adopt GCT under the influence of understanding green prevention and control. Thus, this paper’s first hypothesis is as follows.

**H1.**  *Cooperative’s education positively affect the adoption of GCT by members of cooperatives*.

The cost-benefit theory holds that, as rational economic persons, members will make rapid and correct production decision-making adjustments according to changes in economic returns, and maximize returns by reconfiguring production factors [41]. The theory of “ecological economic man” believes that people in the ecological economic system not only have the economic rationality to pursue the maximization of economic benefits, but also have the ecological rationality to attach importance to the value of the ecological environment [42]. Therefore, members’ decision to adopt GCT is usually based on economic, environmental, and social cognition. If the marginal benefit of an action is greater than the marginal cost, it will increase the implementation of the action, but not vice versa [43]. High-level cognition of GCT among members will lead to a deep understanding of the self-interest, ecology, and altruism of technology adoption [33], so members can more easily perceive the economic, environmental, and social benefits of GCT and cost changes, and then germinate their endogenous motivation to adopt GCT. High-level cognition of GCT can help individuals perceive benefits, and reduce the cost of searching and processing relevant information, and promote efficient acquisition of technology [32]. Therefore, this paper’s second hypothesis is as follows.

**H2.**  *Green prevention and control cognition positively impact GCT adoption by members of cooperatives*.

Cognitive theory believes that the body will generate understanding and views according to the situation and problems it is in, and will concretize and visualize the knowledge of the problem, thereby generating cognition [44]. Cognitive process is a set of information processing systems, including information acquisition, encoding, storage, retrieval, and use of a series of successive stages of cognitive operations [45]. The education of cooperatives expands the channels for members to recognize new green technologies, and promotes the cognition of GCT [34]. In the impact of cooperative’s education on members’ cognition, the acquisition of information is the stimulation directly acting on the senses in the propaganda, the coding of information is the processing of the received green prevention and control information through thinking activities, such as imagination and memory, and the use of information is making a choice based on individual’s cognition of green prevention and control. Theoretically, when cooperatives carry out education, members’ ability to acquire and encode information improves, and their cognition level of GCT increases. The cognition of members can reduce trust costs and technical risks, and improve value perception and profit expectations, thereby promoting members to adopt GCT. According to the logical deduction, this paper proposes the research ideas: Cooperatives’ Education→Members’ Cognition of GCT→Members’ Adoption of GCT.

**Figure 1:**
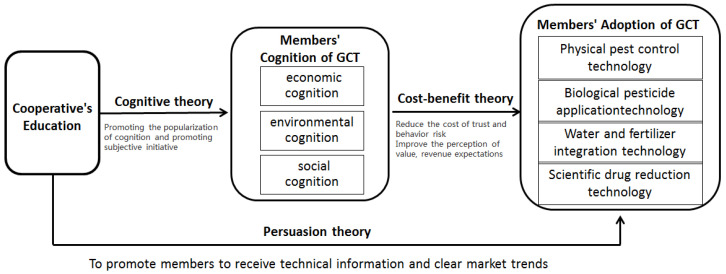
Theoretical model of GCT adopted by members.

Strengthening education in cooperatives can help to improve members’ cognition of GCT, and the improvement of cognitive level can promote the adoption of technology by members. Through logical deduction, it can be seen that education can promote the adoption of GCT through the mediating role of members’ cognition. Accordingly, a theoretical model of GCT adoption by members is constructed, as shown in Figure 1.

Accordingly, Hypothesis H3 is proposed:

**H3.**  *Individuals’ cognition of GCT plays a mediating role in the influence of cooperatives’ education on the GCT adoption of members*.

## 3. Materials and Methods

### 3.1. Data Sources

Because GCT has different technology categories in different agricultural industries, in order to control the endogeneity problem caused by the difference of GCT categories, this paper selects members of citrus planting cooperatives as the research objects. The research data comes from household surveys conducted by the research team in August 2020 and August 2021, covering 14 large counties (districts) for late-ripening citrus cultivation in China. The sampling method is to randomly select 2–4 townships in each sampled county, then randomly select 1–4 farmers’ cooperatives in the selected townships, and then randomly select 5–10 members from the selected cooperatives as the survey objects. A total of 1124 members from 148 cooperatives are selected for this survey. The contents of the survey include education of cooperatives, members’ cognition, GCT adoption, individual characteristics, and family endowments, after statistics and sorting (Table 1).

### 3.2. Variable Selection

#### 3.2.1. Explained Variables

This paper selects four common technologies in the process of citrus planting: physical pest control (yellow plate, insecticidal lamp), biological pesticide application, water and fertilizer integration, and scientific reduction of pesticides use as the representatives of the four categories of GCT. In the questionnaire, the adoption of GCT is represented by whether members adopted four common technologies, and the options for each question included “adopted” and “not adopted”, which are assigned as 1 and 0 respectively. The number of sub-technologies adopted is used as a reflection indicator [46,47], using the sum of four technical adoption assignments to measure the overall adoption of GCT by members. The statistical results show that the proportions of physical pest control, biological pesticide application, water and fertilizer integration, and scientific reduction are 52.2%, 22.9%, 27.8%, and 51.4%, respectively, and the mean of GCT overall adoption is 1.543.

#### 3.2.2. Explanatory Variables

In the questionnaire, “How many times did you receive education on green prevention and control by the cooperative last year?” to represent the education of cooperatives to its members. The statistical results show that the average annual number of sample members receiving cooperative’s education is 4.18.

#### 3.2.3. Mediating Variables

According to the theoretical analysis, this paper divides the green prevention and control cognition of members into three dimensions: economic cognition, environmental cognition, and social cognition. In order to avoid the shortcomings of Delphi method’s strong subjectivity and the limitation of factor analysis method’s emphasis on analyzing quantitative variables, this study uses AHP analytic hierarchy process for weighting. Six experts, including Chinese agricultural economics scholars, agricultural sector personnel, and professional farmers, are invited to score the relative importance of variables at each level according to A.L.Saaty’s 1–9 scale method, and then processed to obtain a discriminant matrix. Next, each variable is weighted to obtain a comprehensive evaluation on the awareness level of members’ green prevention and control, and the specific index settings and weights are shown in Table 2. The statistical results show that the average value of the sample’s cognition of GCT is 3.817.

#### 3.2.4. Control Variables

Referring to existing studies [6,7,8], the control variables mainly include individual characteristics and family endowments. This paper selects 12 variables such as gender, age, and education level of members to control. The specific meaning and assignment of variables are shown in Table 3.

### 3.3. Method

#### 3.3.1. Probit Model and Oprobit Model

The probit model is used to test the influence of cooperative’s education on the GCT adoption of members. The empirical model is set as follows:

(1) $$Prob(Y_{ki}=1|x_{i})=Prob\left(\alpha_{0}education_{i}+\beta_{0}X_{i}+\mu_{0}\right)$$

In Formula (1), $Y_{ki}$ is a binary discrete variable, where $Y_{ki}$ = 1 indicates that the member adopts the GCT, and $Y_{ki}$ = 0 indicates that the member does not use the GCT. As for k, its value from 0 to 4 indicates the adoption and decision-making of four technologies: physical pest control, biological pesticide application, water and fertilizer integration, and scientific reduction of pesticides use. In addition, $publicity_{i}$ represents the ith sample member receiving education of the cooperative; $X_{i}$ is the control variable; $\alpha_{0}$, $\beta_{0}$ are estimated coefficients; $\mu_{0}$ represents the random error term that obeys the standard normal distribution. Since the overall adoption of GCT is an ordered multi-category variable, the Oprobit (Ordered probit) model is used for empirical testing.

The probit model is used to test the impact of green prevention and control cognition on the GCT adoption of members. The empirical model is set as follows:

(2) $$Prob(Y_{ki}=1|x_{i})=Prob\left(\alpha_{1}cognition_{i}+\beta_{1}X_{i}+\mu_{1}\right)$$

In Formula (2), $Y_{ki}$ is a binary discrete variable, where $Y_{ki}$ = 1 means that the member adopts the GCT, and $Y_{ki}$ = 0 means that the member does not use the GCT. The value of k from 0 to 4 represents the adoption decision of the above four technologies, respectively. In addition, $cognition_{i}$ indicates the green prevention and control cognition of the ith sample member; $X_{i}$ is the control variable; $\alpha_{1}$, $\beta_{1}$ are estimated coefficients; $\mu_{1}$ represents the random error term that obeys the standard normal distribution. Similarly, the Oprobit model is used to empirically test the impact of member cognition on the overall adoption of GCT.

#### 3.3.2. Instrumental Variable Method

Farmers’ cognition of a certain aspect and its related behavior may lead to endogeneity problems due to reverse causality and omitted variables. Therefore, this paper adopts the instrumental variable method (IV-Oprobit) to correct the model estimation result and solve the problem of estimation result bias, so as to obtain a consistent and unbiased estimation. Based on the selection condition that instrumental variables should be highly correlated with endogenous explanatory variables, but not related to disturbance terms, this paper selects whether someone around the interviewee adopts GCT as the instrumental variable of the model. There is a strong correlation between members’ cognitive level and the surrounding environment’s perception and acceptance of GCT, but whether or not someone around members adopts GCT is not directly related to members’ own adoption behavior. The selection of this instrumental variable meets the requirements of correlation and exogeneity theoretically [48], and then two regression models are constructed to test it. The results show that this variable has no significant effect on the GCT adoption of members, but is significantly related to members’ cognition, and through the correlation coefficient test, it proves that the setting of the instrumental variable is reasonable.

#### 3.3.3. The Mediation Effect Model

In order to further verify whether the cognition of members plays a significant mediating role between the education of cooperatives and the adoption of GCT, referring to the mediation effect test method [49], the mediation effect model is set as follows:

(3) $$Y_{ki}=\alpha_{0}education_{i}+\beta_{0}X_{i}+\mu_{0}$$

(4) $$cognition_{i}=\alpha_{2}education_{i}+\beta_{2}X_{i}+\mu_{2\;\;\;\;\;\;\;}$$

(5) $$Y_{ki}=\alpha_{3}education_{i}+\beta_{3}cognition_{i}+X_{0}X_{i}+\mu_{3}$$

In Equation (3), $\alpha_{0}$ reflects the total effect of education on GCT adoption of members. In Equation (4), $\alpha_{2}$ represents the effect of education on member cognition as an intermediary variable. In Equation (5), $\alpha_{3}$ and $\beta_{3}\;$ respectively represent the direct effects of education and members’ cognition on the GCT adoption by the i-th member. Substituting Equation (4) into Equation (5) can obtain the mediating effects of members’ cognition, namely $\alpha_{2\;}$ and $\beta_{3}$, that is, the indirect effect of education on GCT adoption through the mediating variable (members’ cognition). At the same time, the ratio of the mediation effect to the total effect is used to reflect the relative size of the mediation effect, namely $\;\alpha_{2}\beta_{3}$/$\alpha_{0}$.

## 4. Results and Discussion

### 4.1. External Driving Role of Education

Table 4 reports the estimated results of the impact of cooperatives’ education on GCT adoption by members. The results of columns (1)–(4) show that education have a significant positive impact on the adoption of physical pest control technologies and biological pesticide application technology at the level of 1%, and positively affect the adoption of water and fertilizer integration technology and scientific pesticides reduction technology at the level of 5%, and the marginal effects of these impacts are 0.063, 0.037, 0.057, and 0.027, respectively. Further, the results in column (5) show that education has a significant positive impact on the overall GCT adoption of members at level of 1%. Thus, hypothesis H1 is confirmed. By carrying out education to popularize green prevention and control knowledge, farmers’ cooperatives directly reduce the marginal cost of members’ technical information search and reception, and promote members’ understanding of the key points of GCT operation. This helps to improve the awareness, knowledge, and ability of members in the green prevention and control of pests and diseases, enhance their professional human capital accumulation in green prevention and control, and then increase their GCT adoption. In conclusion, education have an important external driving effect on GCT adoption of members.

The influence of gender on physical pest control, water and fertilizer integration, scientific pesticides reduction technology, and overall adoption of members is positive and significant at the levels of 10%, 5%, 5%, and 1%, respectively, which is consistent with the fact that women are more likely to receive new information. The influence of age on the adoption of water and fertilizer integration technology by members is only positive and significant at the 5% level, which may be due to the influence of traditional farming methods, farmers are willing to adopt green agricultural technology, but with the increase of age, the enthusiasm of older members to learn new things decreases [50], thus weakening the adoption of GTC. Education at the 1% level promotes physical means of pest control, biopesticide application, and overall GCT adoption by members. Good educational literacy lays a good foundation for members’ own awareness of ecological environmental protection, information reception, and learning ability, and plays an active role in members’ adoption of GCT decision-making, which is consistent with existing research conclusions [51]. The citrus planting area promotes the adoption of integrated water and fertilizer technology and the overall adoption of GCT at the level of 10%. The larger the planting scale, the more likely the members will adopt the ecological regulation-type GCT [52]. Geographic location has a negative and significant impact on the adoption of water and fertilizer integration technology by members at the level of 1%. The long distance between cooperatives and members means that it is difficult for members to receive educational information from the cooperative, thus reducing the possibility of adopting GCT [53]. The influence of topography on the application of biopesticides and the adoption of integrated water and fertilizer technology is negatively significant at the 1% and 10% levels, respectively. Agriculture is highly dependent on natural conditions, and good terrain can provide convenience for technology and reduce costs. Usually, flat terrain and concentrated distribution are favorable for members to adopt GCT [11].

### 4.2. Endogenous Dynamic Effect of Green Prevention and Control Cognition

Table 5 reports the estimated results of the impact of green prevention and control awareness on GCT adoption by members. From the estimation results of instrumental variables in columns (2) and (8), it can be seen that the DWH endogeneity test rejects the null hypothesis that there is no endogenous cognition of members at the level of 5% and 10%, so the regression results of instrumental variables are used to explain. Similarly, the estimation results of instrumental variables in columns (4) and (6) cannot reject the null hypothesis that there is no endogeneity in member cognition, so the benchmark regression results are used for analysis. The F-values estimated in the first stage are all 21.67, indicating that the selected instrumental variables are not weak instrumental variables. The results show that the impact of green prevention and control cognition on members’ adoption of physical pest control, biological pesticide application, water and fertilizer integration, and scientific pesticides reduction technology is positive and significant at the levels of 5%, 5%, 1%, and 5%, respectively. Their marginal effects are 0.800, 0.133, 0.178, and 0.576, respectively. Further, the results in column (10) show that the null hypothesis that there is no endogeneity in member cognition is rejected (atanhrho is significantly different from 0), and green prevention and control cognition improve the overall GCT adoption of members at the level of 1%. Therefore, hypothesis H2 is confirmed. Members with a high level of cognition of GCT have a strong perception of the cost and benefit of adopting technology, which helps to reduce the trust cost and behavioral risk, and improve the value perception and benefit expectation of adoption. Therefore, the initiative and enthusiasm of members to adopt these technology can be fully stimulated.

### 4.3. The Cumulative Effect of Education on Green Prevention and Control Cognition

Table 6 reports the estimated results of the impact of cooperatives’ education on members’ cognition of GCT. The results of columns (1) and (2) both show that the education of cooperatives significantly contributes to the cognition of GCT among members at the level of 1%. From this, it can be seen that the education of green prevention and control knowledge in cooperatives can help improve members’ cognitive ability and understanding of green prevention and control, promote members’ acquisition, coding, and storage of relevant information, and ultimately promote the accumulation of cognition of GCT.

### 4.4. Test of the Mediating Effect of Green Prevention and Control Cognition

Table 7 reports the regression estimation results of introducing the education of cooperatives and the cognition of GCT of members at the same time. According to the DWH test results, the regression results of columns (2), (8), and (10) are used for analysis. In the instrumental variable regression estimation results, the F value of the first stage is 23.54, indicating that there is no weak instrumental variable problem. The results show that after introducing education and cognition of GCT, the cognition of GCT has a positive and significant impact on the adoption of physical pest control technology, biological pesticide application technology, water and fertilizer integration technology, and scientific pesticides reduction technology at the levels of 1%, 1%, 5%, and 10%, respectively, and their marginal effects are 0.715, 0.421, 0.156, and 0.569, respectively. At this time, the influence of education on members’ adoption of physical pest control, biological pesticide application, water and fertilizer integration, and scientific pesticides reduction technology is positive and significant at the level of 5%, 5%, 10%, and 10%, respectively; and their marginal effects are 0.036, 0.024, 0.020, and 0.007, respectively, which are lower than the corresponding marginal effects of education when the members’ green prevention and control cognitive variables are not introduced (0.063, 0.037, 0.057, and 0.027, respectively). Further, the estimation results of the instrumental variables in column (10) show that, rejecting the null hypothesis that there is no endogeneity in the cognition of members, the impact of education on the overall GCT adoption is significant at the 1% level, and the marginal effect of overall adoption at the highest value decreases from 0.057 to 0.046. The above results indicates that members’ cognition plays a partial mediating role in the process of education influencing members’ adoption of physical pest control technology, biological pesticide application technology, water and fertilizer integration technology and scientific pesticides reduction technology. Therefore, hypothesis H3 is confirmed. The education of cooperatives can help improve members’ awareness level of green prevention and control, reduce their trust cost and behavioral risk of adopting GCT, increase the value perception and benefit expectation of behavior, and then stimulate members’ enthusiasm, initiative, and creativity to adopt GCT.

### 4.5. Robustness Check

In order to further ensure the reliability of the research conclusions, this paper conducts a sample robustness test on the main effect model from the aspects of samples and methods.

First, a sample robustness test is performed. Compared with the members who can receive the propaganda and education information more quickly, the older members who are over 80 years old have weaker information ability, and their GCT adoption is weakly related to education. Thus, remove the sample of old members. The obtained result is still significant at the 1% significance level (see Table 8), which shows that the sample has good robustness.

Next, a robustness test of the mediation test method is performed. The test of mediation effect is replaced by Sobel’s method and Bootstrap’s method [54]. The results show that the statistic Z value of the mediating effect test of members’ green prevention and control cognition is 3.61, which is significant at the 1% level. This indicates that member cognition plays a partial mediating role in the relationship between education and the overall adoption of GCT, and the mediating effect accounts for 15.56% (see Table 9). Therefore, the robustness of the mediating role of members’ green prevention and control cognition is confirmed.

### 4.6. Heterogeneity Analysis

Different generations of farmers have different values, cognition, and behavior choices [55]. Combined with the actual situation in rural China, this paper divides the new generation and the old generation of cooperative members according to the 50-year-old boundary, and analyzes the GCT adoption of the two generations of members respectively. It can be seen from Table 10 that among the older generation members, the education of cooperatives has a positive and significant impact on the physical pest control technology, water and fertilizer integration technology, scientific pesticides reduction technology, and overall GCT adoption at the levels of 1%, 1%, 5%, and 1%, respectively. Among the new generation of members, the education of cooperatives has a positive and significant impact on the physical pest control technology, biological pesticide application technology, and the overall adoption of GCT at the level of 1%.The possible explanation is that the older generation of members has a narrower information channel and pays more attention to and trusts the content of the cooperative’s education, while the new generation of members has stronger information receiving and learning abilities, and can recognize the potential benefits of GCT more quickly [50]. The results further verifies that both generations of members can adopt GCT through the external drive of the cooperative’s education.

## 5. Discussion

### 5.1. The Influence of Educational Methods

The above results show that cooperative’s education has an impact on GCT adoption among members. Furthermore, is there any difference in the impact of different education methods on GCT adoption? In theory, different ways of education in cooperatives may lead to different sensory experience of members, which may lead to deviation in their reception and understanding of Green Prevention and control knowledge and difference in their cognition of green production. In general, the more targeted, interactive, and immediate the educational approach is, the more likely it is to deliver relevant ideas and information efficiently to the members, and thus to motivate them to make technology adoption decisions that are more in line with their own perceptions, for better education. Therefore, the way in which cooperatives carry out relevant education may have an impact on the adoption of green prevention and control technologies by their members, this way is divided into the distribution of publicity materials, hold a general meeting of the members, on-the-spot demonstration of publicity, village column and radio propaganda.

The results of columns (1) and (2) in Table 11 show that on the basis of controlling a series of other variables, the four types of education of cooperatives have significant positive effects on the adoption of green prevention and control technologies by members, among them, whether to distribute publicity materials is significant at the 10% confidence level, whether to hold a general meeting for members and whether to conduct on-the-spot demonstration propaganda is significant at the 1% confidence level, the column (3) further validates the above results. The results show that on-the-spot demonstration of cooperative and holding the general meeting of the members are more targeted, interactive, and immediate, the higher the popularization efficiency of green prevention and control knowledge is, the more likely members are to adopt green technology. Through the on-the-spot demonstration and general meetings of the members, the cooperative can be helped to “Find the sticking point” by a good interactive communication environment, and convey information directly, timely and effectively, members are more likely to adopt green prevention and control technologies. In addition, the use of village column and radio propaganda is not targeted, such a way may lead to members of the green prevention and control demand and supply of cooperative actors propaganda objectives are inconsistent, resulting in demand and supply out of line, it is difficult for members to acquire timely and effective green prevention and control knowledge, and the level of cognition and adoption of green prevention and control technology is not high. The propaganda material reading threshold is high, the audience group is small.

### 5.2. Theoretical and Practical Implications

The adoption of a new technology depends on research scientists doing trials and making recommendations, and providing training to the farmers. Similar to previous studies, this paper finds that education of farmers’ cooperatives can effectively promote members to implement green production behavior [22,29,30]. Also confirmed that the positive effect of GCT cognition on adoption [17,20]. However, different from the existing research on education [18,19], this paper focuses on the educational characteristics and effects of cooperative organizations, and innovatively carries out empirical research on the four types of education methods carried out by cooperatives, such as distribution of publicity materials, hold a general meeting of the members, on-the-spot demonstration of publicity, village column and radio propaganda. This paper enriches the application of cognitive theory and cognitive theory, explores the influence mechanism of cooperative education on the members’ green production behavior, and analyzes the best education method, which has great theoretical significance.

Accordingly, this paper puts forward the following policy suggestions: First, establish the education system of cooperatives. Taking cooperatives as an important propaganda subject, encourage them to give full play to their organizational advantages to carry out multi-angle and three-dimensional education on knowledge of green prevention and control, create an atmosphere of green prevention and control in rural society, and externally drive members to adopt GCT. Meanwhile, targeted and flexible education should be adopted based on the characteristics of different intergenerational groups. The second is to improve the awareness level of green prevention and control of members in an all-round way through multiple channels. With the economic, environmental, and social benefits of GCT as the focus of education, cooperatives should continue to stimulate individual subjective initiative, enhance the endogenous motivation of members to actively participate in green prevention and control, and then increase their GCT adoption. Third, guide and encourage cooperatives to adopt targeted and immediate methods of education, such as on-site demonstrations of key members and the holding of general meetings of members, through the implementation of effective ways to enhance the impact of GCT adopted by members, and continuous enrichment and innovation of education methods.

### 5.3. Future Research Directions

Based on the results of this study, two further areas are proposed. On the one hand, the question of how cooperative organizations can promote the change of productive behavior of their members through the dissemination of knowledge is worth studying, as is the impact of the type and manner of knowledge dissemination on green production and the impact mechanisms therein, it will provide a brand-new angle of view for the Cooperative to promote the green production of farmers. We can look at how cooperatives can teach senior extension service staff and then have them train the farmer community. We can also encourage the production of monthly newsletters on technical operations. On the other hand, for farmers, the use of biological pesticides, to avoid or significantly reduce the use of pesticides and other green production behavior is a new thing and system. It can be studied from an economic point of view, comparing the costs and benefits of adoption by farmers and judging whether they are more profitable to adopt green production practices, which will have economic implications.

## 6. Conclusions

This paper systematically explains the correlation mechanism among cooperatives’ education, members’ cognition and GCT adoption, quantitatively examines the effect of education on the four GCTs and the overall adoption of members, and demonstrates the mediating effect of members’ green prevention and control cognition. Empirical studies have found that education have a significant external driving effect on GCT adoption of members, and there is a certain degree of heterogeneity in different generations of members; the improvement of members’ cognitive level can help strengthen their endogenous motivation to adopt GCT. The study further confirms that education can significantly improve members’ cognition of GCT, and can promote members’ adoption of GCT through the partial mediating effect of cognition. Approaches of education also have a significant impact on technology adoption by members, such as distribution of publicity materials and holding a general meeting of the members that can make members more likely to adopt GCT.

## Figures and Tables

**Table 1:** Sample size distribution.

City	County	Sample Size	Percentage/%
Chengdu	Pujiang	60	5.34
	Jintang	74	6.58
Nanchong	Gaoping	69	6.14
	Nanbu	53	4.72
	Pengan	80	7.12
Meishan	Dongpo	60	5.34
	Renshou	115	10.23
	Danling	114	10.14
Ziyang	Anyue	71	6.32
	Yanjiang	119	10.59
Neijiang	Zizhong	113	10.05
Yibin	Jiangan	126	11.20
Dazhou	Dachuan	20	1.78
	Quxian	50	4.45

**Table 2:** Weighted results of green prevention and control cognition.

Variable	Dimensions	Weight	Indicators	Mean	St.d.	Weight
Members’ Cognition of GCT	Economic cognition	0.311 9	GCT can increases citrus production	3.447	0.836	0.078 0
			GCT can raise the price of citrus	3.784	0.821	0.233 9
	Environmental cognition	0.490 5	GCT can improve the environment	4.031	0.691	0.367 9
			GCT can improve soil quality	4.059	0.680	0.122 6
	Social cognition	0.197 6	GCT is good for your health and the health of others	3.970	0.617	0.164 7
			GCT is good for social development	3.938	0.871	0.032 9

**Table 3:** Meaning and assignment of variables.

Variable Type	Variable		Assignment	Mean Value	Standard Deviation	Minimum Value	Maximum Value
**Dependent variable**	Adoption of GCT	Physical pest control	Have = 1; none = 0	0.522	0.500	0	1
		Biological pesticide application	Have = 1; none = 0	0.229	0.420	0	1
		Water and fertilizer integration	Have = 1; none = 0	0.278	0.448	0	1
		Scientific reduction of pesticides	Have = 1; none = 0	0.514	0.500	0	1
		The overall GCT adoption	The sum of the above four assignments	1.543	1.033	0	4
**Independent variable**	Education of farmers’ cooperatives		Number of education campaigns conducted by co-operative societies last year	4.180	3.498	0	30
**Intermediate variable**	Cognition of GCT		The results of cognitive empowerment in three dimensions: economy, environment and society	3.817	0.636	1	5
**Control variable**	Personal characteristics of interviewees	Sex	Female = 1; Male = 0	0.281	0.450	0	1
		Age	Actual age/year	55.565	9.463	25	83
		Level of education	Actual years of education	7.545	3.471	0	18
		Party membership	Party member: Yes = 1; No = 0	0.152	0.359	0	1
		Cadre status	Serving as a cadre of a Village Commune: Yes = 1; No = 0	0.129	0.335	0	1
		Physical fitness	Bad = 1; worse = 2; General = 3; Better = 4; good = 5	4.073	0.800	0	5
		Total household income	Total household income/10,000 ¥ last year	29.410	90.645	0	1500
		Household scale	Citrus acreage/mu	26.405	83.345	0	1200
		Planting time	Years of citrus production /year	11.754	8.935	1	50
	Household Resource Endowment	Geographic location	Distance from home to co-operative/km	1.981	3.052	0	35
		Social Networks	Friends and family working in agricultural systems: Yes = 1; No = 0	0.199	0.589	0	1
		Topography	Village Terrain: Plain = 1; Hill = 2; Mountain = 3	2.038	0.362	1	3

**Table 4:** Estimated impact of cooperative’s education on GCT adoption.

Variable	Physical Pest Control	Biological Pesticide Application	Water and Fertilizer Integration	Scientific Reduction of Pesticides	The Overall GCT Adoption
	Probit (1)	Probit (2)	Probit (3)	Probit (4)	Oprobit (5)
**Education of farmers’ cooperatives**	0.063 *** (0.012)	0.037 *** (0.012)	0.025 ** (0.012)	0.027 ** (0.011)	0.057 *** (0.009)
**Sex**	0.153 * (0.089)	0.005 (0.102)	0.176 ** (0.093)	0.188 ** (0.087)	0.209 *** (0.073)
**Age**	−0.004 (0.005)	0.003 (0.006)	0.014 ** (0.005)	−0.002 (0.005)	0.003 (0.004)
**Level of education**	0.053 *** (0.015)	0.086 *** (0.017)	0.013 (0.015)	−0.004 (0.014)	0.050 *** (0.012)
**Party membership**	0.370 *** (0.129)	0.275 ** (0.128)	0.247 ** (0.127)	0.068 (0.122)	0.367 *** (0.101)
**Cadre status**	0.084 (0.135)	0.030 (0.137)	−0.163 (0.140)	0.170 (0.130)	0.056 (0.108)
**Physical fitness**	0.216 *** (0.053)	0.094 * (0.060)	0.070 (0.056)	0.038 (0.050)	0.155 *** (0.043)
**Total household income**	0.001 (0.000)	0.000 (0.001)	−0.001 (0.001)	−0.000 (0.000)	0.000 (0.000)
**Household scale**	−0.000 (0.001)	0.001 (0.001)	0.001 * (0.001)	0.001 (0.001)	0.001 * (0.000)
**Planting time**	0.006 (0.004)	−0.007 (0.005)	−0.002 (0.005)	0.002 (0.004)	0.001 (0.004)
**Geographic location**	−0.016 (0.013)	−0.009 (0.146)	−0.055 *** (0.014)	−0.006 (0.013)	0.011 (0.011)
**Social Networks**	−0.009 (0.068)	0.064 (0.066)	0.137 * (0.079)	0.051 (0.070)	0.088 * (0.055)
**Topography**	0.119 (0.111)	−0.345 *** (0.130)	−0.200 * (0.114)	−0.099 (0.108)	−0.019 (0.091)
**Sample size**	1124	1124	1124	1124	1124
**LRchi^2^**	134.64 ***	110.59 ***	46.81 ***	21.69 ***	161.56 ***
**Pseudo R^2^**	0.087	0.092	0.035	0.014	0.051

***, ** and * represent the significance level at 1%, 5% and 10% respectively; standard error in parentheses.

**Table 5:** Effect of green prevention and control cognition on GCT adoption.

Variable	Physical Pest Control		Biological Pesticide Application		Water and Fertilizer Integration		Scientific Reduction of Pesticides		The Overall GCT Adoption	
	Probit	IV-Probit	Probit	IV-Probit	Probit	IV-Probit	Probit	IV-Probit	Oprobit	IV-Oprobit
	(1)	(2)	(3)	(4)	(5)	(6)	(7)	(8)	(9)	(10)
**Cognition of GCT**	0.453 *** 0.083	0.800 ** (0.347)	0.133 ** (0.068)	0.751 *** (0.273)	0.178 *** (0.070)	0.435 (0.327)	0.140 ** (0.065)	0.576 ** (0.288)	0.308 *** (0.056)	0.896 *** (0.219)
**Control variable**	under control	under control	under control	under control	under control	under control	Under control	under control	under control	under control
**Sample size**	1124	1124	1124	1124	1124	1124	1124	1124	1124	1124
**LRchi^2^/wald chi^2^**	109.70 ***	129.48 ***	133.17 ***	115.75 ***	49.07 ***	43.45 ***	20.74 ***	22.10 ***	155.32 ***	641.59 ***
**FIRST-STAGE F**		21.67 ***		21.67 ***		21.67 ***		21.67 ***		
**Dwh Test**		3.952 **		0.808		0.751		2.21 *		
**atanhrho**										−0.377 ***
**Pseudo R^2^**	0.071		0.110		0.037		0.013		0.049	

***, ** and * represent the significance level at 1%, 5% and 10% respectively; standard error in parentheses.

**Table 6:** Estimated results of the impact of cooperative’s education on members’ cognition of GCT.

	OLS (1)	Oprobit (2)
**Education of farmers’ cooperatives**	0.032 *** (0.005)	0.060 *** (0.009)
**Control variable**	under control	under control
**Sample size**	1124	1124
**F/LRchi^2^**	20.99 ***	235.07 ***
**R^2^/Pseudo R^2^**	0.197	0.022

***, ** and * represent the significance level at 1%, 5% and 10% respectively; standard error in parentheses.

**Table 7:** Test of the mediating effect of green prevention and control cognition.

Variable	Physical Pest Control		Biological Pesticide Application		Water and Fertilizer Integration		Scientific Reduction of Pesticides		The Overall GCT Adoption	
	Probit	IV-Probit	Probit	IV-Probit	Probit	IV-Probit	Probit	IV-Probit	Oprobit	IV-Oprobit
	(1)	(2)	(3)	(4)	(5)	(6)	(7)	(8)	(9)	(10)
**Education of** **farmers’ cooperatives**	0.061 *** (0.123)	0.036 ** (0.018)	0.024 ** (0.126)	0.012 (0.018)	0.020 * (0.012)	0.011 (0.017)	0.023 ** (0.012)	0.007 * (0.016)	0.050 *** (0.010)	0.046 *** (0.009)
**Cognition of GCT**	0.065 (0.069)	0.715 *** (0.289)	0.421 *** (0.084)	0.777 ** (0.363)	0.156 ** (0.071)	0.413 (0.343)	0.117 * (0.067)	0.569 * (0.301)	0.257 *** (0.057)	0.842 *** (0.223)
**Control variable**	under control	under control	under control	under control	under control	under control	under control	under control	under control	under control
**Sample size**	1124	1124	1124	1124	1124	1124	1124	1124	1124	1124
**LRchi^2^/wald chi^2^**	135.51 ***	154.97 ***	136.87 ***	122.39 ***	51.70 ***	47.13 ***	24.76 ***	27.74 ***	182.35 ***	671.05 ***
**FIRST-** **STAGE F**		23.54 ***		23.54 ***		23.54 ***		23.54 ***		
**Dwh Test**		3.886 **		0.772		0.724		2.156 *		
**atanhrho**										−0.372 ***
**Pseudo R^2^**	0.087		0.113		0.039		0.016		0.057	

***, ** and * represent the significance level at 1%, 5% and 10% respectively; standard error in parentheses.

**Table 8:** Sample stability tests.

Variable	Physical Pest Control	Biological Pesticide Application	Water and Fertilizer Integration	Scientific Reduction of Pesticides	The Overall GCT Adoption
	Probit (1)	Probit (2)	Probit (3)	Probit (4)	Oprobit (5)
**Education of farmers’ cooperatives**	0.064 *** (0.012)	0.038 *** (0.012)	0.025 ** (0.012)	0.027 ** (0.011)	0.057 *** (0.009)
**Control variable**	under control	under control	under control	Under control	under control
**Sample size**	1122	1122	1122	1122	1122
**LRchi^2^**	135.57 ***	112.38 ***	47.09 ***	22.05 ***	162.64 ***
**Pseudo R^2^**	0.087	0.093	0.036	0.014	0.051

***, ** and * represent the significance level at 1%, 5% and 10% respectively; standard error in parentheses.

**Table 9:** Bootstrap tests for mediating effects.

	Total Effect	Mesomeric Effect	Percentage	BootSE	LLCI	ULCI
**members’ cognition of GCT**	0.045	0.007	0.132	0.002	0.003	0.011

**Table 10:** Effects of cooperative’s education on GCT adoption of members from different generations.

Variable	Physical Pest Control		Biological Pesticide Application		Water and Fertilizer Integration		Scientific Reduction of Pesticides		The Overall GCT Adoption	
	Probit		Probit		Probit		Probit		Oprobit	
	Older Generation	New Generation	Older Generation	New Generation	Older Generation	New Generation	Older Generation	New Generation	Older Generation	New Generation
**Education of farmers’ coopera-tives**	0.061 *** (0.014)	0.069 *** (0.024)	0.024 (0.015)	0.073 *** (0.023)	0.029 *** (0.014)	0.013 (0.024)	0.026 ** (0.014)	0.022 (0.022)	0.056 *** (0.011)	0.060 *** (0.018)
**Control variable**	under control	under control	under control	under control	under control	under control	under control	under control	under control	under control
**Sample size**	785	339	785	339	785	339	785	339	785	339
**LRchi^2^**	106.41 ***	32.45 ***	75.66 ***	45.77 ***	28.73 ***	34.69 ***	16.32 ***	30.52 ***	109.01 ***	54.64 ***
**Pseudo R^2^**	0.098	0.071	0.097	0.110	0.031	0.088	0.015	0.065	0.051	0.054

***, ** and * represent the significance level at 1%, 5% and 10% respectively; standard error in parentheses.

**Table 11:** Impact of ways in which cooperatives promote education on members’ adoption of GCT.

Variable	Oprobit (1)		Oprobit (2)		OLS (3)	
	Coefficient	Standard Error	Coefficient	Standard Error	Coefficient	Standard Error
**Distribution of publicity materials**	0.330 ***	0.114	0.224 *	0.119	0.204 **	0.104
**Holding a general meeting of the members**	0.420 ***	0.111	0.392 ***	0.113	0.342 ***	0.100
**On-the-spot demonstration of publicity**	0.486 ***	0.103	0.377 ***	0.108	0.319 ***	0.094
**Village column and Radio Propaganda**	−0.066	0.119	−0.041	0.127	−0.035	0.112
**Control variable**	no control		under control		under control	
**R^2^/Pseudo R^2^**	0.031		0.053		0.135	
**F/LRchi^2^**	52.37 ***		90.43 ***		5.83 ***	

***, ** and * represent the significance level at 1%, 5% and 10% respectively.

## Data Availability

The data presented in this study are available within the article.

## References

[1] Jin J., Wang W., He R., Gong H. (2017). Pesticide use and risk perceptions among small scale farmers in Anqiu County, China. Int. J. Environ. Res. Public Health.

[2] Grung M., Lin Y., Zhang H., Steen A.O., Huang J., Zhang G., Larssen T. (2015). Pesticide levels and environmental risk in aquatic environments in China: A review. Environ. Int..

[3] Gao Y., Niu Z., Yang H., Yu L. (2019). Impact of Green Control Techniques on Family Farms Welfare. Ecol. Econ..

[4] Gao Y., Zhang X., Lu J., Wu L., Yin S. (2017). Adoption Behavior of Green Control Techniques by Family Farms in China: Evidence from 676 Family Farms in Huang-Huai-Hai Plain. Crop Prot..

[5] Yin S., Li R., Wu L., Chen X. (2018). Introduction to 2018 China Development Report on Food Satety.

[6] Abdollahzadeh G., Sharifzadeh M.S., Damalas C.A. (2015). Perceptions of the beneficial and harmful effects of pesticides among Iranian rice farmers influence the adoption of biological control. Crop Prot..

[7] Bola A., Aziz A., Aliou D. (2016). Agricultural technology adoption, commercialization and smallholder rice farmers’ welfare in rural Nigeria. Agric. Food Econ..

[8] Gao Y., Li P., Wu L., Lu J., Yu L., Yin S. (2018). Preferences of for-profit pest control firms on support policy in China. J. Clean. Prod..

[9] Erbaugh J.M., Donnermeyer J., Amujal M., Kidoido M. (2010). Assessing the impact of farmer field school participation on IPM Adoption in uganda. J. Int. Agric. Ext. Educ..

[10] Kassie M., Jaleta M., Shiferaw B., Mmbando F., Mekuria M. (2013). Adoption of interrelated sustainable agricultural practices in smallholder systems: Evidence from rural Tanzania. Technol. Forecast. Soc. Change.

[11] Irawan E. (2016). Adoption model of falcataria-based farm forestry: A duration analysis approach. J. Ekon. Pembang..

[12] Chattopadhyay P., Banerjee G., Mukherjee S. (2017). Recent trends of modern bacterial insecticides for pest control practice in integrated crop management system. 3 Biotech.

[13] Benelli G., Pavela R., Maggi F., Petrelli R., Nicoletti M. (2017). Commentary: Making green pesticides greener? The potential of plant products for nanosynthesis and pest control. J. Clust. Sci..

[14] Baloch A., Thapa B. (2014). Agricultural extension in Balochistan, Pakistan: Date palm farmers’ access and satisfaction. J. Mt. Sci..

[15] Marsh L., Zoumenou V., Cotton C., Hashem F. (2017). Organic farming: Knowledge, practices, and views of limited resource farmers and nonfarmers on the Delmarva Peninsula. Org. Agric..

[16] Hoque B.A., Hallman K., Levy J., Bouis H., Ali N., Khan F., Khanam S., Kabir M., Hossain S., Alam M.S. (2006). Rural drinking water at supply and household levels: Quality and management. Int. J. Hyg. Environ. Health.

[17] Ajzen I. (2011). The theory of planned behaviour: Reactions and reflections. Psychol. Health.

[18] Yazdanpanah M., Feyzabad F.R. (2017). Investigating Iranian farmers’ satisfaction with agricultural extension programs using the American customer satisfaction index. J. Agric. Food Inf..

[19] Timprasert S., Datta A., Ranamukhaarachchi S. (2014). Factors determining adoption of integrated pest management by vegetable growers in Nakhon Ratchasima Province Thailand. Crop Prot..

[20] Onumah J.A., Williams P.A., Quaye W., Akuffobea M., Onumah E.E. (2014). Smallholder cocoa farmers access to on/off-farm support services and its contribution to output in the eastern region of Ghana. Asian J. Agric. Rural. Dev..

[21] Latynskiy E., Berger T. (2016). Networks of rural producer organizations in Uganda: What can be done to make them work better?. World Dev..

[22] Elahi E., Abid M., Zhang L., Ul Haq S., Sahito J.G.M. (2018). Agricultural advisory and financial services; farm level access, outreach and impact in a mixed cropping district of Punjab, Pakistan. Land Use Policy.

[23] Mujawamariya G., D’Haese M., Speelman S. (2013). Exploring double side selling in cooperatives, case study of four coffee cooperatives in Rwanda. Food Policy.

[24] Benjamin E.O., Blum M., Punt M. (2016). The impact of extension and ecosystem services on smallholder’s credit constraint. J. Dev. Areas.

[25] Ochilo W.N., Otipa M., Oronje M., Chege F., Lingeera E.K., Lusenaka E., Okonjo E.O. (2018). Pest management practices prescribed by frontline extension workers in the smallholder agricultural subsector of Kenya. J. Integr. Pest Manag..

[26] Villamil M.B., Alexander M., Silvis A.H., Gray M.E. (2012). Producer perceptions and information needs regarding their adoption of bioenergy crops. Renew. Sustain. Energy Rev..

[27] Tregear A., Cooper S. (2016). Embeddedness, social capital and learning in rural areas: The case of producer cooperatives. J. Rural Stud..

[28] Francesconi G.N., Wouterse F. (2018). Building the Managerial Capital of Agricultural Cooperatives in Africa. Ann. Public Coop. Econ..

[29] Huang J., Huang Z., Jia X., Hu R., Xiang C. (2015). Long-term reduction of nitrogen fertilizer use through knowledge training in rice production in China. Agric. Syst..

[30] Yu L., Chen C., Niu Z., Gao Y., Yang H., Xue Z. (2020). Risk Aversion, Cooperative Membership and the Adoption of Green Control Techniques: Evidence from China. J. Clean. Prod..

[31] Sharma R., Peshin R. (2016). Impact of integrated pest management of vegetables on pesticide use in subtropical Jammu, India. Crop Prot..

[32] Bukchin S., Kerret D. (2018). Food for hope: The role of personal resources in farmers’ adoption of green technology. Sustainability.

[33] Stern P.C., Dietz T., Guagnano G.A. (1995). The new ecological paradigm in social-psychological context. Environ. Behav..

[34] Bourdieu P. (1986). The Forms of Capital.

[35] Pan D., Zhang N. (2018). The role of agricultural training on fertilizer use knowledge: A randomized controlled experiment. Ecol. Econ..

[36] Atanu S., Love H.A., Schwart R., Saha A. (1994). Adoption of emerging technologies under output uncertainty. Am. J. Agric. Econ..

[37] Hovland C., Janis I., Kelley H. (1953). Communication and persuasion. Audiov. Commun. Rev..

[38] Ma W., Abdulai A. (2018). IPM adoption, cooperative membership and farm economic performance. China Agric. Econ. Rev..

[39] Spielman D.J., Byerlee D., Alemu D., Kelemework D. (2010). Policies to promote cereal intensification in Ethiopia: The search for appropriate public and private roles. Food Policy.

[40] Zhang S., Sun Z., Ma W., Valentinov V. (2020). The effect of cooperative membership on agricultural technology adoption in Sichuan, China. China Econ. Rev..

[41] Dowlatshahi S. (2010). A cost-benefit analysis for the design and implementation of reverse logistics systems: Case studies approach. Int. J. Prod. Res..

[42] Gintis H. (2000). Beyond homo economicus: Evidence from experimental economics. Ecol. Econ..

[43] Falcon W., Schultz T. (1988). Transforming Traditional Agriculture. Am. J. Agric. Econ..

[44] Bandura A. (1986). Social Foundations of Thought and Action.

[45] Mathews A., MacLeod C. (1994). Cognitive Approaches to Emotion and Emotional Disorders. Annu. Rev. Psychol..

[46] Korir J., Affognon H., Ritho C., Kingori W., Irungu P., Mohamed S., Ekesi S. (2015). Grower adoption of an integrated pest management package for management of mango-infesting fruit flies (Diptera: Tephritidae) in Embu, Kenya. Int. J. Trop. Insect Sci..

[47] Allahyari M.S., Damalas C.A., Ebadattalab M. (2016). Determinants of integrated pest management adoption for olive fruit fly (*Bactrocera oleae*) in Roudbar, Iran. Crop Prot..

[48] Staiger D., Stock J.H. (1997). Instrumental variables regression with weak instruments. Econometrica.

[49] MacKinnon D., Fairchild A., Fritz M. (2007). Mediation Analysis. Annu. Rev. Psychol..

[50] Hussian M., Zia S., Saboor A. (2011). The adoption of integrated pest management (IPM) technologies by cotton growers in the Punjab. Soil Environ..

[51] Gao Y., Zhang X., Wu L., Yin S., Lu J. (2017). Resource basis, ecosystem and growth of grain family farm in China: Based on rough set theory and hierarchical linear model. Agric. Syst..

[52] Cockburn J., Coetzee H., Van den Berg J., Conlong D. (2014). Large-scale sugarcane farmers’ knowledge and perceptions of *Eldana saccharina* walker (Lepidoptera: Pyralidae), push-pull and integrated pest management. Crop Prot..

[53] Zhou J., Liu Q., Liang Q. (2018). Cooperative membership, social capital, and chemical input use: Evidence from China. Land Use Policy.

[54] Wooldridge J. (2010). Econometric Analysis of Cross-Section and Panel Data.

[55] Lyons S., Kuron L. (2014). Generational differences in the workplace: A review of the evidence and directions for future research. J. Organ. Behav..

